# Prospective genetic profiling of squamous cell lung cancer and adenosquamous carcinoma in Japanese patients by multitarget assays

**DOI:** 10.1186/1471-2407-14-786

**Published:** 2014-10-28

**Authors:** Hirotsugu Kenmotsu, Masakuni Serizawa, Yasuhiro Koh, Mitsuhiro Isaka, Toshiaki Takahashi, Tetsuhiko Taira, Akira Ono, Tomohiro Maniwa, Shoji Takahashi, Keita Mori, Masahiro Endo, Masato Abe, Isamu Hayashi, Takashi Nakajima, Yasuhisa Ohde, Nobuyuki Yamamoto

**Affiliations:** Division of Thoracic Oncology, Shizuoka Cancer Center, 1007 Shimonagakubo, Nagaizumi-cho, Sunto-gun, Shizuoka, 411-8777 Japan; Division of Drug Discovery and Development, Shizuoka Cancer Center Research Institute, Nagaizumi-cho, Sunto-gun, Japan; Division of Thoracic Surgery, Shizuoka Cancer Center, Nagaizumi-cho, Sunto-gun, Japan; Clinical Trial Coordination Office, Shizuoka Cancer Center, Nagaizumi-cho, Sunto-gun, Japan; Division of Diagnostic Radiology, Shizuoka Cancer Center, Nagaizumi-cho, Sunto-gun, Japan; Division of Pathology, Shizuoka Cancer Center, Nagaizumi-cho, Sunto-gun, Japan; Third Department of Internal Medicine, Wakayama Medical University, Kimiidera, Wakayama, Japan

**Keywords:** Lung cancer, Squamous cell carcinoma, Adenosquamos carcinoma, Genetic profiling, Driver mutation, *PIK3CA* mutation, *FGFR1* copy number gain

## Abstract

**Background:**

Despite considerable recent progress in the treatment of lung adenocarcinoma, there has been little progress in the development of efficacious molecular targeted therapies for squamous cell lung cancer. In addition to the recent comprehensive genome-wide characterization of squamous cell lung cancer, it is also important to genotype this form of cancer. We therefore conducted the Shizuoka Lung Cancer Mutation Study to analyze driver mutations in patients with thoracic malignancies. Here we report the results of genotyping in patients with squamous cell lung cancer.

**Methods:**

Based on the biobanking system, in conjunction with the clinic and pathology lab, we developed a genotyping panel designed to assess 24 mutations in 10 genes (*EGFR*, *KRAS*, *BRAF*, *PIK3CA*, *NRAS*, *MEK1*, *AKT1*, *PTEN*, *HER2* and *DDR2*), *EGFR*, *MET*, *PIK3CA*, *FGFR1* and *FGFR2* copy numbers, and *EML4-ALK* and *ROS1* translocations, using pyrosequencing plus capillary electrophoresis, quantitative polymerase chain reaction (PCR) and reverse-transcription PCR, respectively.

**Results:**

A total of 129 patients with squamous cell lung cancer and adenosquamous carcinoma were enrolled in this study between July 2011 and November 2012. We detected genetic alterations in 40% of all cases. Gene alterations included: *EGFR* mutations, 6%; *KRAS* mutations, 4%; *PIK3CA* mutations, 13%; *NRAS* mutations, 1%; *KIF5b-RET* fusion gene, 1%; *EGFR* copy number gain, 5%; *PIK3CA* copy number gain, 15%; and *FGFR1* copy number gain, 5%. Twelve patients (9%) harbored simultaneous genetic alterations. Genetic alterations were detected more frequently in surgically-resected, snap-frozen samples than in formalin-fixed, paraffin-embedded samples (50% vs. 29%). In addition, patients aged ≤70 years old and never-smokers showed high frequencies of genetic alterations.

**Conclusions:**

This study represents one of the largest prospective tumor-genotyping studies to be performed in Asian patients with squamous cell lung cancer. These results suggest that incorporation of genetic profiling into lung cancer clinical practice may facilitate the administration of personalized cancer treatments in patients with squamous cell lung cancer.

**Electronic supplementary material:**

The online version of this article (doi:10.1186/1471-2407-14-786) contains supplementary material, which is available to authorized users.

## Background

Non-small-cell lung cancer (NSCLC) has recently been divided into nonsquamous cell carcinoma and squamous cell carcinoma. Pemetrexed and bevacizumab have been approved for the treatment of nonsquamous cell lung cancer [1, 2]. In addition, epidermal growth factor receptor (*EGFR*) mutations and anaplastic lymphoma kinase (*ALK*) fusion genes have been identified in lung adenocarcinoma, and are considered as biomarkers for EGFR and ALK inhibitors [3–7]. Treatment for nonsquamous cell lung cancer has therefore advanced, including options for personalized therapy.

Squamous cell lung cancer is a major histological subtype of NSCLC, accounting for 30% of NSCLC. However, in contrast to adenocarcinomas, little progress has been achieved in the development of efficacious molecular targeted therapies for squamous cell lung cancer. Comprehensive genome-wide characterization of squamous cell lung cancer has recently revealed some potential drug targets [8–10]. However, differences in frequencies of some genetic alterations, including *EGFR* and *KRAS* mutations, have been identified between Asian and Western patients [11], and it is therefore important to assess the frequencies of genetic alterations in squamous cell lung cancer in different ethnic groups, including in Asian patients.

We developed a tumor-genotyping panel to screen lung cancer patients for genetic alterations relevant to novel molecular-targeted therapeutics in ongoing clinical trials [12–15] (Additional file 1: Table S1). Genotyping analysis was implemented in the Shizuoka Lung Cancer Mutation Study, which is a prospective tumor-genotyping study conducted in patients admitted to Shizuoka Cancer Center with thoracic malignancies. This paper reports the results of this study in relation to genetic alterations in squamous cell lung cancer and adenosquamous carcinoma.

## Methods

### Patients and samples

The Shizuoka Lung Cancer Mutation Study was initiated in July 2011 to analyze driver mutations in patients with thoracic malignancies. The study subjects were patients with pathologically-diagnosed thoracic malignancies, who had provided written informed consent. The diagnosis and differentiation of squamous cell carcinoma and adenosquamous carcinoma were confirmed by institutional pathologists, in accordance with the 2004 World Health Organization classification. When samples were difficult to diagnose as squamous cell carcinoma, immunohistochemical analyses were performed (i.e., thyroid transcription factor 1, p63 staining). Surgically-resected tissue specimens were macrodissected by the same pathologists to enrich the tumor content. Tumor biopsy specimens containing ≥10% tumor content, as evaluated by hematoxylin-eosin staining, were used for this study. All specimens from 129 patients with squamous cell lung cancer were thus considered adequate for genotyping. Surgically-resected tissues were snap-frozen on dry ice immediately after resection and stored at -80°C until use. Formalin-fixed, paraffin-embedded (FFPE) specimens, mainly including biopsy samples, were sectioned at a thickness of 10 μm. All the relevant clinicopathological information, including smoking history, was retrieved from the patients’ medical records. We defined “light smokers” as those who smoked <30 packs per year, and “heavy smokers” as those who smoked ≥30 packs per year.

### Genetic profiling

We developed a tumor genotyping panel (Table 1) to assess 24 hot-spot sites of genetic alterations in 10 genes (*EGFR*, *KRAS*, *BRAF*, *PIK3CA*, *NRAS*, *MEK1*, *AKT1*, *PTEN, HER2* and *DDR2*), *EGFR*, *MET*, *PIK3CA*, *FGFR1* and *FGFR2* copy number gains, and *EML4-ALK*, *KIF5B-RET*, *CCDC6-RET, CD74-ROS1* and *SLC34A2-ROS1* fusion genes using pyrosequencing plus capillary electrophoresis, quantitative polymerase chain reaction (PCR), and reverse-transcription PCR, respectively. These genetic alterations were selected based on the articles listed in Additional file 1: Table S1. Detailed methods are described in Additional file 2 [16]. Fusion genes were accessed only with fresh-frozen tissues.

**Table 1:** **Multiple tumor genotyping panel**

Gene	Position	AA mutant	Nucleotide mutant
*EGFR*	G719	G719C/S	2155G > T/A
		G719A	2156G > C
	exon 19	deletion	
	T790	T790M	2369C > T
	exon 20	insertion	
	L858	L858R	2573 T > G
	L861	L861Q	2582 T > A
*KRAS*	G12	G12C/S/R	34G > T/A/C
		G12V/A/D	35G > T/C/A
	G13	G13C/S/R	37G > T/A/C
		G13D/A	38G > A/C
	Q61	Q61K	181C > A
		Q61R/L	182A > G/T
		Q61H	183A > T/C
*BRAF*	G466	G466V	1397G > T
	G469	G469A	1406G > C
	L597	L597V	1789C > G
	V600	V600E	1799 T > A
*PIK3CA*	E542	E542K	1624G > A
	E545	E545K/Q	1633G > A/C
	H1047	H1047R	3140A > G
*NRAS*	Q61	Q61K	181C > A
		Q61L/R	182A > T/G
*MEK1 (MAP2K1)*	Q56	Q56P	167A > C
	K57	K57N	171G > T
	D67	D67N	199G > A
*AKT1*	E17	E17K	49G > A
*PTEN*	R233	R233*	697C > T
*HER2*	exon 20	insertion	
*DDR2*	S768	S768R	2304 T > A

### Statistical analysis

All categorical variables were analyzed by *χ*^2^ or Fisher’s exact tests, as appropriate. All p values were reported as two-sided, and values <0.05 were considered statistically significant. This study was approved by the Institutional Review Board of the Shizuoka Cancer Center (22-34-22-1-7).

## Results

### Patient characteristics

A total of 129 patients were diagnosed with squamous cell lung cancer or adenosquamous carcinoma and were included in this study from July 2011 to November 2012. The characteristics of the patients are shown in Table 2. The median age was 70 years (range: 38–92), and most patients were male and smokers. Histologically, adenosquamous carcinoma was observed in six (5%) of the patients. Well-differentiated, moderately-differentiated and poorly-differentiated squamous cell carcinomas were present in 10%, 53% and 27% of the patients, respectively. Stage I, II, III and IV were observed in 26%, 29%, 26% and 19%, respectively. Surgically-resected, snap-frozen samples were obtained from 64 patients (50%), and FFPE samples from 65 patients (50%).

**Table 2:** **Patient characteristics (overall, n =129)**

	N =129	(%)
Median age (years)	70	
(range)	(38–92)	
Gender		
Male	111	86
Female	18	14
Smoker		
Never	3	2
Light (pack-year <30)	12	9
Heavy (pack-year ≥30)	114	89
Histology		
Squamous	123	95
Adenosquamous	6	5
Differentiation		
Well	13	10
Moderately	69	53
Poorly	35	27
Unknown	6	5
Stage		
I	33	26
II	38	29
III	34	26
IV	24	19

### Genetic alteration profiles

**Figure 1:**
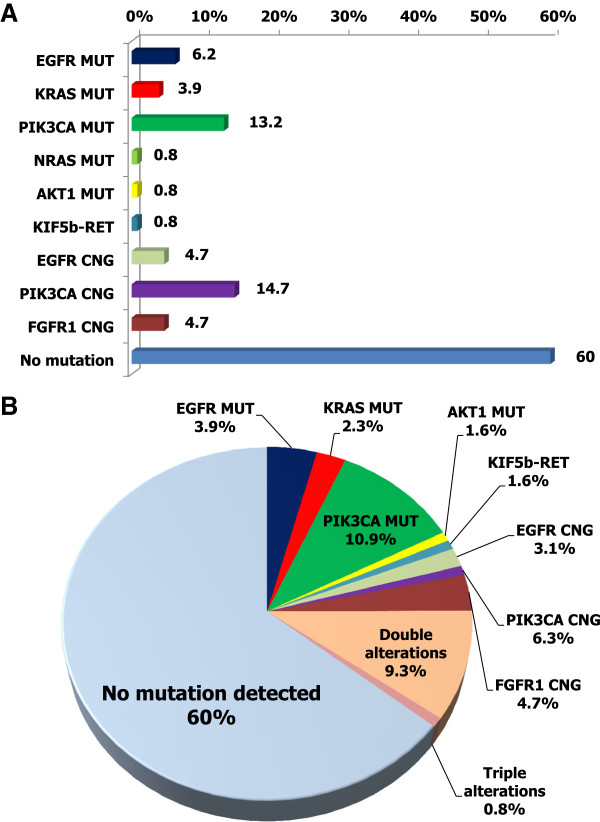
**Relative proportions of genetic alterations in squamous cell lung cancer and adenosquamous carcinoma (overall, n = 129). A**: Pie chart shows relative proportions of genetic alterations. **B**: Bar chart shows relative proportions of genetic alterations. MUT: mutant, CNG: copy number gain.

**Figure 2:**
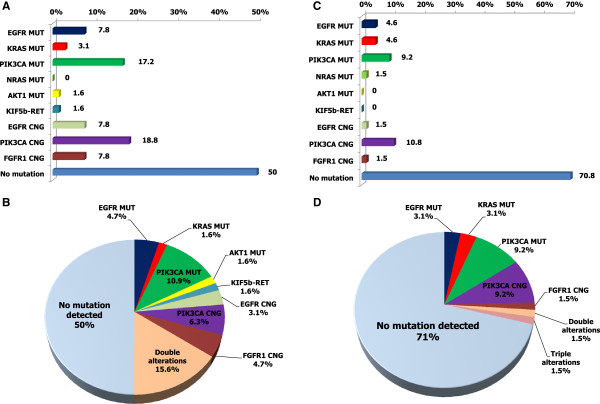
**Relative proportions of genetic alterations in surgically resected snap-frozen samples (A and B, n = 64) and paraffin-embedded samples (C and D, n = 65) from patients with squamous cell lung cancer and adenosquamous carcinoma. A**: Bar chart shows relative proportions of genetic alterations in surgically resected snap-frozen samples. **B**: Pie chart shows relative proportions of genetic alterations in surgically resected snap-frozen samples. **C**: Bar chart shows relative proportions of genetic alterations in paraffin-embedded samples. **D**: Pie chart shows relative proportions of genetic alterations in paraffin-embedded samples. MUT: mutant, CNG: copy number gain.

We detected genetic alterations in 40% of all cases. Figure 1 shows the frequencies of genetic alterations in patients with squamous cell lung cancer. The genetic alterations included: *EGFR* mutation in eight (6%); *KRAS* mutation in five (4%); *PIK3CA* mutation in 17 (13%); *NRAS* mutation in one (1%); *KIF5b-RET* fusion in one (1%); *EGFR* copy number gain in six (5%); *PIK3CA* copy number gain in 19 (15%); and *FGFR1* copy number gain in six (5%) (Additional file 3: Table S2 and Additional file 4: Table S3). Of eight patients with *EGFR* mutation, four had the L858R point mutation in exon 21, and three had deletions in exon 19. In addition, the frequencies of genetic alterations in surgically-resected, snap-frozen samples and FFPE samples from patients with squamous cell lung cancer were analyzed (Figure 2), and the following alterations were detected: *EGFR* mutation in 8% and 5%, *KRAS* mutation in 3% and 5%, *PIK3CA* mutation in 17% and 9%, *EGFR* copy number gain in 8% and 2%, *PIK3CA* copy number gain in 19% and 11%, and *FGFR1* copy number gain in 8% and 2%, respectively.

### Clinicopathological factors related to genetic alterations

The results of univariate analysis of clinicopathological factors for genetic alterations are shown in Table 3. Genetic alterations were significantly more frequent in surgically-resected, snap-frozen samples than in FFPE samples (50% vs. 29%, p = 0.015). In addition, patients ≤70 years old and “never-smokers” showed higher frequencies of genetic alterations. Also, 75% of patients ≤60 years old (n = 12) had genetic alterations including *EGFR* mutation in 2, *KRAS* mutation in 2, *PIK3CA* mutation in 2, *KIF5b-RET* fusion in 1, *EGFR* copy number gain in 2, and *PIK3CA* copy number gain in 2.

**Table 3:** **Frequency of genomic alterations in clinicopathological factors (overall, n =129)**

	Genomic alterations		p value
	(+)	(-)	
Age			0.027
≤70 years	33	35	
>70 years	18	48	
Gender			N.S.
Male	44	67	
Female	7	11	
Smoker			0.035
Never	3	10	
Light (pack-year <30)	3	9	
Heavy (pack-year ≥30)	45	69	
Histology			N.S.
Squamous	49	74	
Adenosquamous	2	4	
Differentiation			N.S.
Well	5	8	
Moderately	27	42	
Poorly	15	20	
Unknown	2	4	
Stage			N.S.
I	13	20	
II	12	26	
III	16	18	
IV	10	14	
Samples			0.015
Snap-frozen	32	32	
FFPE	19	46	

*FFPE* formalin-fixed paraffin-embedded.

## Discussion

This study represents one of the largest, prospective, tumor-genotyping studies carried out in Asian patients with squamous cell carcinoma of the lung. Genetic alterations were detected in 40% of patients in this study. There have been few reports on the gene alterations associated with squamous cell lung cancer. However, the Cancer Genome Atlas Research Network performed a comprehensive genomic analysis of 178 squamous cell lung cancers and reported the following genetic alterations: *PIK3CA* mutations in 16%, *PTEN* mutation/deletion in 15%, *FGFR1* amplification in 15%, *EGFR* amplification in 9%, *PDGFRA* amplification in 9%, *DDR2* mutation in 4%, and unknown genetic alterations in 21% [8]. In addition, multiplex testing for driver mutations in 72 squamous cell carcinomas of the lung detected: *PIK3CA* mutations in 8%, *PTEN* mutation/deletion in 28%, *FGFR1* amplification in 26%, and unknown genetic alterations in 39% [9]. Korean study showed a similar spectrum of gene alterations between East Asian and North American [10]. Genetic alterations in patients enrolled in the current prospective study may reflect the frequencies of genetic alterations in the clinical setting, and suggest that genetic profiling in Japanese patients may be similar to that in North American.

Genetic alterations were seen more frequently in surgically-resected, snap-frozen samples, in patients ≤70 years old, and in “never-smokers”. FFPE specimens are subject to increasing DNA degradation as they get older [17], which may account for the difference in frequencies of genetic alterations between snap-frozen and FFPE samples. Squamous cell lung cancer is strongly associated with cigarette smoking [18] and 98% of patients with squamous cell carcinoma in this study were light or heavy smokers. Although all three “never-smokers” showed genetic alterations (*EGFR* mutation, *EGFR* or *PIK3CA* copy number gain), the sample size was too small to evaluate these results. The association between age and genetic alterations is unclear. Multiple genetic alterations were reported to be more common in younger patients with papillary thyroid cancer [19], while younger patients with colorectal cancer showed a high frequency of KRAS mutations [20]. In contrast however, a positive association between *EGFR* mutation and age was reported among never-smoker lung cancer patients [21].

In this study, *PIK3CA* mutation was relatively frequent in squamous cell lung cancer, as reported in other studies, while *FGFR1* copy number gain seemed less frequent [8, 9]. The phosphoinositide 3-kinase (PI3K) pathway is a key oncogenic signaling pathway that functions in cell survival and proliferation [22]. The *PIK3CA* gene encodes the PI3K catalytic subunit α-isoform and is frequently mutated in some of the most common human tumors. Our earlier study, as well as other studies, found that *PIK3CA* mutations were more common in squamous cell lung cancer than in lung adenocarcinoma [23–25]. The fibroblast growth factor receptor (FGFR) is a transmembrane receptor tyrosine kinase that participates in the regulation of embryonal development, cell proliferation, differentiation, and angiogenesis [26, 27]. The frequency of *FGFR1* amplification in surgical specimens has been reported to be 13–41%, and does not seem to differ according to ethnicity [28–30]. However, the frequency of *FGFR1* copy number gain in this study was only 4% of all samples and 8% of fresh-frozen samples. This apparent discrepancy in the frequencies of *FGFR1* copy number gain may be a result of the different methodologies used in the studies, and/or the influence of biopsy samples from patients with metastatic squamous cell lung cancer. *PIK3CA* mutation and *FGFR1* amplification both represent potential targets for personalized squamous cell lung cancer therapy, and it may therefore be important to analyze both these gene alterations in clinical practice.

A major limitation of this study was that genetic alterations were analyzed using a genotyping panel, rather than by a comprehensive analysis. However, the objective of this study was not only to assess the frequencies of driver gene mutations, but also to assign patients to appropriate therapies and/or enrollment in clinical trials. Our genotyping panel included most gene mutations that are targeted by new drugs in ongoing clinical trials. This study was also limited by intratumor heterogeneity, which may have resulted in underestimation of tumor genetic alterations [31]. It is difficult to obtain multiple lesions by tumor biopsy in the clinical setting, but we intend to address this challenge in the future to aid further progress in biomarker development.

## Conclusion

Genetic alterations were detected in 40% of Japanese patients with squamous cell lung cancer. These results suggest that incorporation of genetic profiling into lung cancer clinical practice may facilitate the administration of personalized cancer treatments in patients with squamous cell lung cancer, though further studies are needed to verify these results.

## Electronic supplementary material

Additional file 1: Table S1: Tumor genotyping panel developed for this study. (PDF 243 KB)

Additional file 2: **Supplementary methods.** (PDF 129 KB)

Additional file 3: Table S2: Distribution of genetic alterations in each gene. (PDF 106 KB)

Additional file 4: Table S3: Distribution of concurrent genetic alterations. (PDF 21 KB)
