# Supplementary material for: Prospective genetic profiling of squamous cell lung cancer and adenosquamous carcinoma in Japanese patients by multitarget assays
Source: BMC Cancer. 2014 Oct 28;14:786. doi: 10.1186/1471-2407-14-786 (PMC4221703; doi:10.1186/1471-2407-14-786)
Supplement: Supplementary file 1 — Additional file 1: Table S1: Tumor genotyping panel developed for this study. (PDF 243 KB) [file 12885_2014_4965_MOESM1_ESM.pdf]

Table S1. Tumor genotyping panel developed for this study

Gene Name	Type [1]	Position	Amino acid mutant	Nucleotide mutant	Positive control cell lines	Method [2]	PCR primer [3, 4] Forward	Reverse	Sequence	Amplicon Length (bp) [5]	Reference
EGFR	SNV	G719	G719C/S	2155G>T/A	-	P	TCCCAACCAAGCTCTCTTGAGG	*CTGTGCCAGGGACCTTACCTTATA	AAAAAGATCAAAGTGCTG	109	[1-9]
			G719A	2156G>C							
	Deletion	exon 19			PC-9 (E746_A750del), HCC827 (E746_A750del), HCC4006 (L747_E749del)	F	TCCCAGAAGGTGAGAAAGTTAAAA	CACAGCAAAGCAGAAACTCACAT		110	
	SNV	T790	T790M	2369C>T	NCI-H1975	P	CTGGGCATCTGCCTCACCT	*GTCTTTGTGTTCCCGGACAT	CCGTGCAGCTCATCA	90	
	Insertion	exon 20			-	F	CATGCGAAGCCACACTGAC	GAGGCAGATGCCCAGCAG		102	
	SNV	L858	L858R	2573T>G	NCI-H1975	P	GAAAACACCGCAGCATGTCA	*TTTGCCTCCTTCTGCATGGTA	AAGATCACAGATTTTGG	91	
	SNV	L861	L861Q	2582T>A	-						
	CNG				HCC827, A431	qPCR	GAATTCGGATGCAGAGCTTC	GACATGCTGCGGTGTTTTC		150	[10]
KRAS	SNV	G12	G12C/S/R	34G>T/A/C	A549 (G12S)	P	GGCCTGCTGAAAATGACTGAA	*TTAGCTGTATCGTCAAGGCACTCT	TGTGGTAGTTGGAGCT	82	[1-4, 11, 12]
			G12V/A/D	35G>T/C/A	NCI-H441 (G12V)						
	SNV	G13	G13C/S/R	37G>T/A/C	-						
			G13D/A	38G>A/C	DLD-1 (G13D)						
	SNV	Q61	Q61K	181C>A	-	P	AATTGATGGAGAAACCTGTCTCTT	*TTATGGCAAATACACAAAGAAAGC	CTCGACACAGCAGGT	119	
			Q61R/L	182A>G/T	-						
			Q61H	183A>T/C	NCI-H1155 (183A>T)						
BRAF	SNV	G466	G466V	1397G>T	NCI-H1666	P	GAGATTCCTGATGGGCAGATTA	*TACATACTTACCATGCCACTTTCC	GACAAAGAATTGGATCTG	93	[1-4, 13-15]
	SNV	G469	G469A	1406G>C	NCI-H1395, NCI-H1755						
	SNV	L597	L597V	1789C>G	-	P	AAGACCTCACAGTAAAAATAGGTG	*AAAATGGATCCAGACAACTGTTC	AAAAATAGGTGATTTTGGT	97	
	SNV	V600	V600E	1799T>A	HT29, COLO201						
PIK3CA	SNV	E542	E542K	1624G>A	IM95	P	*GAACAGCTCAAAGCAATTTCTACA	TAGCACTTACCTGTGACTCCATAG	AGAAAATCTTTCTCCTGCT	90	[1-4, 13, 14, 16-18]
	SNV	E545	E545K/Q	1633G>A/C	DLD-1 (E545K)						
	SNV	H1047	H1047R	3140A>G	-	P	*AGCAAGAGGCTTTGGAGTATTTC	GTTCAATGCATGCTGTTTAATTGT	GTTGTCCAGCCACCA	110	
	CNG				Calu3, NCI-H520	qPCR	ATCTTTTCTCAATGATGCTTGGCT	CTAGGGTCTTTCGAATGTATG		81	[17-19]
NRAS	SNV	Q61	Q61K	181C>A	-	P	TGGTGAAACCTGTTTGTTGGACAT	*TGGTCTCTCATGGCACTGTACTCT	ATACTGGATACAGCTGGA	69	[1-3, 20]
			Q61L/R	182A>T/G	HepG2 (Q61L)						
MEK1	SNV	Q56	Q56P	167A>C	NCI-H1437	P	ATGAGCAGCAGCGAAAGC	*AGCCCCCAGCTCACTGATC	GAGGCCTTTCTTACCC	101	[1, 3, 13, 21]
	SNV	K57	K57N	171G>T	-						
	SNV	D67	D67N	199G>A	ES-2	P	*GCCTTGAGGCCTTTCTTACCC	ACACCACACCGCCATTGC	CTCACTGATCTTCTCAAAGT	102	

Supplementary Table S1. Tumor genotyping panel developed for this study (*continuation*)

Gene Name	Type [1]	Position	Amino acid mutant	Nucleotide mutant	Positive control cell lines	Method [2]	PCR primer [3]				Reference
							Forward	Reverse	Sequence	Amplicon Length (bp) [4]	
AKT1	SNV	E17	E17K	49G>A	-	P	CTGACGGGTAGAGTGT GCG	***GCCGCCAGGTCTTGAT GTA**	CGCACGTCTGTA GGG	70	[1, 3, 13, 22]
PTEN	SNV	R233	R233*	697C>T	HCI-H1155	P	TTTGTGGTCTGCCAGCT AAAG	***TAACGGCTGAGGGAA CTCAAA**	CAATTCAGGACC CACA	99	[2]
DDR2	SNV	S768	S768R	2304T>A	-	P	***ATAGGGCAAGTTCACT ACAGCAA**	ATAGGGCTGTTCTTGAC AAAAGG	CAAAGGCCCACA CAT	88	[23]
HER2	Insertion	exon 20			NCI-H1781 (G776insVC)	F	CTCAGCGTACCCTTGT CCC	CTGCACCGTGGATGTC AG		100	[1-4, 13, 14, 24, 25]
MET	CNG				EBC-1, NCI-H2170	qPCR	GCTGGTGGTCCTACCA TACATG	CTGGCTTACAGCTAGTT TGCCA		112	[1, 4, 13, 26]
FGFR1	CNG				Calu3, NCI-H1703	qPCR	TTCCTCATCTCCTGCAT GGT	GTGGTGCTGAGTGTGC AAAT		167	[4, 14, 27]
FGFR2	CNG				SNU-16, KATOIII	qPCR	ACTTGGGCTGGAGTGA TTTG	AATCCCATCTGCACACT TCC		164	[27]
COL8A1	Control for copy number analysis					qPCR	GGGCTAAGAAAGGCAA GAATGG	GTGGGAAAGGTGCGGT TAGCT		78	[18]
LINE1	Control for copy number analysis					qPCR	AAAGCCGCTCAACTAC ATGG	TGCTTTGAATGCGTCCC AGAG		149	-
ALK	Fusion	*EML4-ALK* (variant 1, ,2, 3a/b, 4, 5a/b, 6, 7)			NCI-H2228 (variant 3)	RT	TGATGTTTTGAGGCGTC TTG<br>TTAGCATTCTTGGGGAA TGG	TGCCAGCAAAGCAGTA GTTG		-	[1, 2, 4, 13, 28, 29]
ROS1	Fusion	*CD74-ROS1*, *SLC34A2-ROS1*			-	RT	CCTGAGACACCTTAAG AACACCA<br>TCGGATTTCTCTACTTT TTCGTG	TGAAACTTGTTTCTGGT ATCCAA		-	[4, 14, 30, 31]
RET	Fusion	*KIF5B-RET* (variant 1, 2, 3, 4, 5, 6, 8), *CCDC6-RET*			LC-2/ad (*CCDC6-RET*)	RT	Primers and methods for detecting *KIF5B-RET* and *CCDC6-RET* fusion genes were kindly provided by Dr. Takashi Kohno (National Cancer Center, Tokyo, Japan). Therefore, we cannot disclose primer information.				[4, 14, 32-35]
GAPDH	Control for reverse transcription PCR					RT	GCACCGTCAAGGCTGA GAAC	TGGTGAAGACGCCAGT GGA		138	-

[1] SNV, Single nucleotide variation, CNG, Copy number gain

[2] P, Pyrosequencing; F, Fragment analysis; qPCR, Quantitative PCR; RT, Reverse Transcription PCR

[3] Bold and *, 5' Biotinylated primers

[4] Amplicon length of fusion genes in each variants were not described.

References

1. Johnson BE, Kris MG, Kwiatkowski DJ, Lafrate AJ, Wistuba II, Shyr Y, A. EJ, Khuri FR, M. DS, Pao W *et al*: **Clinical characteristics of planned 1000 patients with adenocarcinoma of lung (ACL) undergoing genomic characterization in the US Lung Cancer Mutation Consortium (LCMC)**. *J Thorac Oncol 2011; 6 (suppl 2; abstr O1601)*.
2. Sequist LV, Heist RS, Shaw AT, Fidias P, Rosovsky R, Temel JS, Lennes IT, Digumarthy S, Waltman BA, Bast E *et al*: **Implementing multiplexed genotyping of non-small-cell lung cancers into routine clinical practice**. *Ann Oncol* 2011, **22**(12):2616-2624.
3. Su Z, Dias-Santagata D, Duke M, Hutchinson K, Lin YL, Borger DR, Chung CH, Massion PP, Vnencak-Jones CL, Iafrate AJ *et al*: **A platform for rapid detection of multiple oncogenic mutations with relevance to targeted therapy in non-small-cell lung cancer**. *J Mol Diagn* 2011, **13**(1):74-84.
4. Gadgeel SM: **New targets in non-small cell lung cancer**. *Curr Oncol Rep* 2013, **15**(4):411-423.
5. Lynch TJ, Bell DW, Sordella R, Gurubhagavatula S, Okimoto RA, Brannigan BW, Harris PL, Haserlat SM, Supko JG, Haluska FG *et al*: **Activating mutations in the epidermal growth factor receptor underlying responsiveness of non-small-cell lung cancer to gefitinib**. *N Engl J Med* 2004, **350**(21):2129-2139.
6. Paez JG, Janne PA, Lee JC, Tracy S, Greulich H, Gabriel S, Herman P, Kaye FJ, Lindeman N, Boggon TJ *et al*: **EGFR mutations in lung cancer: correlation with clinical response to gefitinib therapy**. *Science* 2004, **304**(5676):1497-1500.
7. Pao W, Miller V, Zakowski M, Doherty J, Politi K, Sarkaria I, Singh B, Heelan R, Rusch V, Fulton L *et al*: **EGF receptor gene mutations are common in lung cancers from "never smokers" and are associated with sensitivity of tumors to gefitinib and erlotinib**. *Proc Natl Acad Sci U S A* 2004, **101**(36):13306-13311.
8. Ohashi K, Maruvka YE, Michor F, Pao W: **Epidermal growth factor receptor tyrosine kinase inhibitor-resistant disease**. *J Clin Oncol* 2013, **31**(8):1070-1080.
9. Rho JK, Choi YJ, Lee JK, Ryoo BY, Na, II, Yang SH, Lee SS, Kim CH, Yoo YD, Lee JC: **The role of MET activation in determining the sensitivity to epidermal growth factor receptor tyrosine kinase inhibitors**. *Molecular cancer research : MCR* 2009, **7**(10):1736-1743.
10. Pirker R, Pereira JR, von Pawel J, Krzakowski M, Ramlau R, Park K, de Marinis F, Eberhardt WE, Paz-Ares L, Storkel S *et al*: **EGFR expression as a predictor of survival for first-line chemotherapy plus cetuximab in patients with advanced non-small-cell lung cancer: analysis of data from the phase 3 FLEX study**. *Lancet Oncol* 2012, **13**(1):33-42.
11. Brose MS, Volpe P, Feldman M, Kumar M, Rishi I, Gerrero R, Einhorn E, Herlyn M, Minna J, Nicholson A *et al*: **BRAF and RAS mutations in human lung cancer and melanoma**. *Cancer Res* 2002, **62**(23):6997-7000.
12. Ogino S, Kawasaki T, Brahmandam M, Yan L, Cantor M, Namgyal C, Mino-Kenudson M, Lauwers GY, Loda M, Fuchs CS: **Sensitive sequencing method for KRAS mutation detection by Pyrosequencing**. *J Mol Diagn* 2005, **7**(3):413-421.
13. Pao W, Girard N: **New driver mutations in non-small-cell lung cancer**. *Lancet Oncol* 2011, **12**(2):175-180.
14. Oxnard GR, Binder A, Janne PA: **New targetable oncogenes in non-small-cell lung cancer**. *J Clin Oncol* 2013, **31**(8):1097-1104.
15. Paik PK, Arcila ME, Fara M, Sima CS, Miller VA, Kris MG, Ladanyi M, Riely GJ: **Clinical characteristics of patients with lung adenocarcinomas harboring BRAF**

mutations. *J Clin Oncol* 2011, **29**(15):2046-2051.

16. Kawano O, Sasaki H, Endo K, Suzuki E, Haneda H, Yukiue H, Kobayashi Y, Yano M, Fujii Y: **PIK3CA mutation status in Japanese lung cancer patients**. *Lung Cancer* 2006, **54**(2):209-215.
17. Okudela K, Suzuki M, Kageyama S, Bunai T, Nagura K, Igarashi H, Takamochi K, Suzuki K, Yamada T, Niwa H *et al*: **PIK3CA mutation and amplification in human lung cancer**. *Pathol Int* 2007, **57**(10):664-671.
18. Yamamoto H, Shigematsu H, Nomura M, Lockwood WW, Sato M, Okumura N, Soh J, Suzuki M, Wistuba, II, Fong KM *et al*: **PIK3CA mutations and copy number gains in human lung cancers**. *Cancer Res* 2008, **68**(17):6913-6921.
19. Spoerke JM, O'Brien C, Huw L, Koeppen H, Fridlyand J, Brachmann RK, Haverty PM, Pandita A, Mohan S, Sampath D *et al*: **Phosphoinositide 3-kinase (PI3K) pathway alterations are associated with histologic subtypes and are predictive of sensitivity to PI3K inhibitors in lung cancer preclinical models**. *Clin Cancer Res* 2012, **18**(24):6771-6783.
20. Ohashi K, Sequist LV, Arcila ME, Lovly CM, Chen X, Rudin CM, Moran T, Camidge DR, Vnencak-Jones CL, Berry L *et al*: **Characteristics of lung cancers harboring NRAS mutations**. *Clin Cancer Res* 2013, **19**(9):2584-2591.
21. Marks JL, Gong Y, Chitale D, Golas B, McLellan MD, Kasai Y, Ding L, Mardis ER, Wilson RK, Solit D *et al*: **Novel MEK1 mutation identified by mutational analysis of epidermal growth factor receptor signaling pathway genes in lung adenocarcinoma**. *Cancer Res* 2008, **68**(14):5524-5528.
22. Bleeker FE, Felicioni L, Buttitta F, Lamba S, Cardone L, Rodolfo M, Scarpa A, Leenstra S, Frattini M, Barbareschi M *et al*: **AKT1(E17K) in human solid tumours**. *Oncogene* 2008, **27**(42):5648-5650.
23. Hammerman PS, Sos ML, Ramos AH, Xu C, Dutt A, Zhou W, Brace LE, Woods BA, Lin W, Zhang J *et al*: **Mutations in the DDR2 kinase gene identify a novel therapeutic target in squamous cell lung cancer**. *Cancer discovery* 2011, **1**(1):78-89.
24. Shigematsu H, Takahashi T, Nomura M, Majmudar K, Suzuki M, Lee H, Wistuba, II, Fong KM, Toyooka S, Shimizu N *et al*: **Somatic mutations of the HER2 kinase domain in lung adenocarcinomas**. *Cancer Res* 2005, **65**(5):1642-1646.
25. De Greve J, Teugels E, Geers C, Decoster L, Galdermans D, De Mey J, Everaert H, Umelo I, In't Veld P, Schallier D: **Clinical activity of afatinib (BIBW 2992) in patients with lung adenocarcinoma with mutations in the kinase domain of HER2/neu**. *Lung Cancer* 2012, **76**(1):123-127.
26. Engelman JA, Zejnullahu K, Mitsudomi T, Song Y, Hyland C, Park JO, Lindeman N, Gale CM, Zhao X, Christensen J *et al*: **MET amplification leads to gefitinib resistance in lung cancer by activating ERBB3 signaling**. *Science* 2007, **316**(5827):1039-1043.
27. Dienstmann R, Rodon J, Prat A, Perez-Garcia J, Adamo B, Felip E, Cortes J, Iafrate AJ, Nuciforo P, Tabernero J: **Genomic aberrations in the FGFR pathway: opportunities for targeted therapies in solid tumors**. *Ann Oncol* 2013.
28. Soda M, Choi YL, Enomoto M, Takada S, Yamashita Y, Ishikawa S, Fujiwara S, Watanabe H, Kurashina K, Hatanaka H *et al*: **Identification of the transforming EML4-ALK fusion gene in non-small-cell lung cancer**. *Nature* 2007, **448**(7153):561-566.
29. Sun Y, Ren Y, Fang Z, Li C, Fang R, Gao B, Han X, Tian W, Pao W, Chen H *et al*: **Lung adenocarcinoma from East Asian never-smokers is a disease largely**

defined by targetable oncogenic mutant kinases. *J Clin Oncol* 2010, **28**(30):4616-4620.

30. Bergethon K, Shaw AT, Ou SH, Katayama R, Lovly CM, McDonald NT, Massion PP, Siwak-Tapp C, Gonzalez A, Fang R *et al*: **ROS1 rearrangements define a unique molecular class of lung cancers**. *J Clin Oncol* 2012, **30**(8):863-870.
31. Li C, Fang R, Sun Y, Han X, Li F, Gao B, Iafrate AJ, Liu XY, Pao W, Chen H *et al*: **Spectrum of oncogenic driver mutations in lung adenocarcinomas from East Asian never smokers**. *PLoS One* 2011, **6**(11):e28204.
32. Kohno T, Ichikawa H, Totoki Y, Yasuda K, Hiramoto M, Nammo T, Sakamoto H, Tsuta K, Furuta K, Shimada Y *et al*: **KIF5B-RET fusions in lung adenocarcinoma**. *Nat Med* 2012, **18**(3):375-377.
33. Takeuchi K, Choi YL, Togashi Y, Soda M, Hatano S, Inamura K, Takada S, Ueno T, Yamashita Y, Satoh Y *et al*: **KIF5B-ALK, a novel fusion oncokinase identified by an immunohistochemistry-based diagnostic system for ALK-positive lung cancer**. *Clin Cancer Res* 2009, **15**(9):3143-3149.
34. Lipson D, Capelletti M, Yelensky R, Otto G, Parker A, Jarosz M, Curran JA, Balasubramanian S, Bloom T, Brennan KW *et al*: **Identification of new ALK and RET gene fusions from colorectal and lung cancer biopsies**. *Nat Med* 2012, **18**(3):382-384.
35. Ju YS, Lee WC, Shin JY, Lee S, Bleazard T, Won JK, Kim YT, Kim JI, Kang JH, Seo JS: **A transforming KIF5B and RET gene fusion in lung adenocarcinoma revealed from whole-genome and transcriptome sequencing**. *Genome Res* 2012, **22**(3):436-445.
