# Supplementary material for: Prospective genetic profiling of squamous cell lung cancer and adenosquamous carcinoma in Japanese patients by multitarget assays
Source: BMC Cancer. 2014 Oct 28;14:786. doi: 10.1186/1471-2407-14-786 (PMC4221703; doi:10.1186/1471-2407-14-786)
Supplement: Supplementary file 2 — Additional file 2: Supplementary methods. (PDF 129 KB) [file 12885_2014_4965_MOESM2_ESM.pdf]

Prospective genetic profiling of squamous cell lung cancer and adenosquamous carcinoma in Japanese patients by multitarget assays

Kenmotsu *et al.*

SUPPLEMENTARY METHODS

Nucleic acid sample preparation

Genomic DNAs were extracted from surgically-resected tissues and/or tumor biopsies using a QIAamp DNA mini kit (Qiagen, Hilden, Germany). A QIAamp DNA FFPE tissue kit (Qiagen) was used to extract genomic DNAs from formalin-fixed, paraffin-embedded (FFPE) samples. DNA concentrations were determined using spectrophotometry (NanoDrop 2000C; Thermo Scientific, Wilmington, DE, USA) and adjusted to 10 ng/μl. The criterion for DNA purity was an optical density $(OD)_{260}/OD_{280}$ ≥1.8. Quantitative polymerase chain reaction (qPCR) was performed to determine gene copy number using a double-strand DNA quantification kit (Quant-iT PicoGreen dsDNA Assay kit, Invitrogen, Carlsbad, CA, USA) to quantify DNA concentration. DNA concentration was adjusted to 1 ng/μl. Total RNAs were extracted from surgically-resected, fresh-frozen tissues using an RNeasy Mini kit (Qiagen) following

standard protocols, and quantified by spectrophotometry (NanoDrop 2000C; Thermo Scientific). RNAs indicating OD_{260}/OD_{280} ≥1.8 were used for the detection of fusion genes.

Pyrosequencing for detection of single-nucleotide variations

Pyrosequencing was used to detect single-nucleotide variations (SNVs) in nine genes (*EGFR*, *KRAS*, *BRAF*, *PIK3CA*, *NRAS*, *MEK1*, *AKT1*, *PTEN* and *DDR2*) (Supplementary Table S1). An internal fragment of each gene was amplified by PCR using a PyroMark PCR Kit (Qiagen) with 20 ng of genomic DNA and primers specific for each genomic region. PCR products were sequenced using a PyroMark Q24 pyrosequencer (Qiagen) with PyroMark Gold Q96 Reagents (Qiagen) and sequencing primers specific for each genomic region. Cell lines used as positive controls are shown in Table S4, and wild-type cell lines were used as negative controls.

Fragment-size analysis to detect insertion/deletion-type genetic alterations

Three insertion/deletion-type genetic alterations in *EGFR* and *HER2* (Supplementary Table S1) were determined by sizing PCR-amplified products using capillary electrophoresis (QIAxcel Advanced System; Qiagen) with the QIAxcel DNA High

Resolution Kit (Qiagen). PCR was performed with 20 ng of genomic DNA, primers specific for each genomic region and a PyroMark PCR Kit (Qiagen). Cell lines used as positive controls are shown in Table S4, and wild-type cell lines were used as negative controls.

Gene copy number analysis

The copy numbers of five genes (*EGFR*, *MET*, *PIK3CA*, *FGFR1* and *FGFR2*; Supplementary Table S1) were determined using qPCR with SYBR green, using a StepOnePlus Real-time PCR system (Applied Biosystems, Foster City, CA) using 2 ng genomic DNA, PCR primers for each gene and SYBR Premix Ex Taq II (Tli RNaseH Plus) (Takara Bio, Shiga, Japan). Target gene copies were quantified by generating standard calibration curves using serial dilutions (10^2–10^8 copies) of recombinant plasmid DNA for each gene, using plasmids constructed in the pCR2.1-TOPO vector (Invitrogen). The copy number of each gene was normalized using the copy number of *LINE-1*. Gene copy number changes were determined by calculating the ratio of the normalized quantity of the target gene to that of *COL8A1*. Results that were ≥2-fold higher than the average value in negative control cell lines and human genome DNAs (Clontech, Palo Alto, CA; Promega, Madison, WI, USA) were considered to show gene

copy number gain. DNAs extracted from the following cell lines with copy number gain in each gene were used as positive controls: *EGFR* (HCC827 [1], A431 [2]), *MET* (EBC-1 [1], NCI-H2170 [1]), *PIK3CA* (Calu3 [3], NCI-H520 [4]), *FGFR1* (Calu3 [5], NCI-H1703 [5]) and *FGFR2* (SNU-16 [6], KATOIII [6]).

Detection of fusion genes

ALK, *ROS1*, and *RET* fusions (Supplementary Table S1) were detected by reverse-transcription (RT)-PCR using RNA from fresh-frozen samples. cDNA templates were synthesized with total RNA (1 μg), random primers (hexadeoxyribonucleotide mixture; pd$(N)_6$) (Takara Bio) and an Omniscript RT Kit (Qiagen). *GAPDH* expression was used as a positive control in RT-PCR reactions. Detection of *EML4-ALK* and *ROS1* fusion genes (*CD74-ROS1* and *SLC34A2-ROS1*) was performed according to the techniques developed by Sun *et al.*[7] and Li *et al.*[8], respectively. Information on the primers and methods for detecting *KIF5B-RET* and *CCDC6-RET* fusion genes were kindly provided by Dr. Takashi Kohno (National Cancer Center, Tokyo, Japan).

REFERENCES

1. McDermott U, Sharma SV, Dowell L, et al. Identification of genotype-correlated

sensitivity to selective kinase inhibitors by using high-throughput tumor cell line profiling. *Proc Natl Acad Sci U S A*. 2007;104: 19936-19941.

2. Moroni M, Veronese S, Benvenuti S, et al. Gene copy number for epidermal growth factor receptor (EGFR) and clinical response to antiEGFR treatment in colorectal cancer: a cohort study. *Lancet Oncol*. 2005;6: 279-286.

3. Yamamoto H, Shigematsu H, Nomura M, et al. PIK3CA mutations and copy number gains in human lung cancers. *Cancer Res*. 2008;68: 6913-6921.

4. Spoerke JM, O'Brien C, Huw L, et al. Phosphoinositide 3-kinase (PI3K) pathway alterations are associated with histologic subtypes and are predictive of sensitivity to PI3K inhibitors in lung cancer preclinical models. *Clin Cancer Res*. 2012;18: 6771-6783.

5. Dutt A, Ramos AH, Hammerman PS, et al. Inhibitor-sensitive FGFR1 amplification in human non-small cell lung cancer. *PLoS One*. 2011;6: e20351.

6. Matsumoto K, Arao T, Hamaguchi T, et al. FGFR2 gene amplification and clinicopathological features in gastric cancer. *Br J Cancer*. 2012;106: 727-732.

7. Sun Y, Ren Y, Fang Z, et al. Lung adenocarcinoma from East Asian never-smokers is a disease largely defined by targetable oncogenic mutant kinases. *J Clin Oncol*. 2010;28: 4616-4620.

8. Li C, Fang R, Sun Y, et al. Spectrum of oncogenic driver mutations in lung adenocarcinomas from East Asian never smokers. *PLoS One*. 2011;6: e28204.

9. Takeuchi K, Choi YL, Togashi Y, et al. KIF5B-ALK, a novel fusion oncokinase identified by an immunohistochemistry-based diagnostic system for ALK-positive lung cancer. *Clin Cancer Res*. 2009;15: 3143-3149.
