# Supplementary material for: Prospective genetic profiling of squamous cell lung cancer and adenosquamous carcinoma in Japanese patients by multitarget assays
Source: BMC Cancer. 2014 Oct 28;14:786. doi: 10.1186/1471-2407-14-786 (PMC4221703; doi:10.1186/1471-2407-14-786)
Supplement: Supplementary file 3 — Additional file 3: Table S2: Distribution of genetic alterations in each gene. (PDF 106 KB) [file 12885_2014_4965_MOESM3_ESM.pdf]

Table S2. Distribution of genetic alterations in each gene

Gene Name[a]	Genetic alterations identified	n	(%)
EGFR (8, 6.2%)			
	ex19del	3	
	L858R	4	
	L858R + T790M		
	ex20ins		
	ex19del + T790M		
	ex19del + L858R		
	G719S		
	G719A	1	
	L861Q	2	
	ex19del + L858R + T790M	1	
	G719S + L861Q + T790M	1	
	Gene copy number gain	6	
KRAS (5, 3.9%)			
	G12C	2	
	G12D		
	G12A		
	G12V	1	
	Q61H	1	
	G12S	1	
	G12F		
PIK3CA (17, 13.2%)			
	E545K	11	
	H1047R	3	
	E545Q	1	
	E542K	2	
	Gene copy number gain	19	
NRAS (1, 0.8%)			
	Q61R	1	
	Q61K		
AKT1 (1, 0.8%)			
	E17K	1	
FGFR1 (6, 4.7%)			
	Gene copy number gain	6	
RET (1/66, 1.5 %)			
	KIF5B-RET variant 2	1	

[a]Numbers in parentheses next to gene names indicate the number and frequency of patients who harbor genetic alterations in each gene.
