# Supplementary material for: Prospective genetic profiling of squamous cell lung cancer and adenosquamous carcinoma in Japanese patients by multitarget assays
Source: BMC Cancer. 2014 Oct 28;14:786. doi: 10.1186/1471-2407-14-786 (PMC4221703; doi:10.1186/1471-2407-14-786)
Supplement: Supplementary file 4 — Additional file 4: Table S3: Distribution of concurrent genetic alterations. (PDF 21 KB) [file 12885_2014_4965_MOESM4_ESM.pdf]

## Table S3. Distribution of concurrent genetic alterations

| Type of concurrent genetic alterations identified | n | % |
|---|---|---|
| *EGFR* mutation + *NRAS* mutation | 1 | (0.8) |
| *EGFR* mutation + *EGFR* copy number gain | 1 | (0.8) |
| *EGFR* mutation + *PIK3CA* copy number gain | 1 | (0.8) |
| *KRAS* mutation + *PIK3CA* mutation | 1 | (0.8) |
| *PIK3CA* mutation + *PIK3CA* copy number gain | 3 | (2.4) |
| *PIK3CA* copy number gain + *EGFR* copy number gain | 2 | (1.6) |
| *PIK3CA* copy number gain + F*GFR1* copy number gain | 2 | (1.6) |
| *KRAS* mutation + *EGFR* copy number gain + *PIK3CA* copy number gain | 1 | (0.8) |
